# HER-2 Expression in Immunohistochemistry Has No Prognostic Significance in Gastric Cancer Patients

**DOI:** 10.1100/2012/941259

**Published:** 2012-05-03

**Authors:** Agnieszka Halon, Piotr Donizy, Przemyslaw Biecek, Julia Rudno-Rudzinska, Wojciech Kielan, Rafal Matkowski

**Affiliations:** ^1^Department of Pathomorphology, Wroclaw Medical University, ul. Marcinkowskiego 1, 50-368 Wroclaw, Poland; ^2^Faculty of Mathematics, Informatics and Mechanics, University of Warsaw, Krakowskie Przedmieście 26/28, 00-927 Warsaw, Poland; ^3^Department of General and Oncological Surgery, Wroclaw Medical University, ul. Borowska 213, 50-556 Wroclaw, Poland; ^4^Department of Oncology, Wroclaw Medical University, Plac Hirszfelda 12, 53-413 Wroclaw, Poland; ^5^2nd Department of Surgical Oncology, Lower Silesian Oncology Center–Regional Comprehensive Cancer Center, Plac Hirszfelda 12, 53-413 Wroclaw, Poland

## Abstract

The role of HER-2 expression as a prognostic factor in gastric cancer (GC) is still controversial. The aim of the study was to asses HER-2 status, its correlations with clinicopathological parameters, and prognostic impact in GC patients. Tumor samples were collected from 78 patients who had undergone curative surgery. In order to evaluate the intensity of immunohistochemical (IHC) reactions two scales were applied: the immunoreactive score according to Remmele modified by the authors and standardised Hercep test score modified for GC by Hofmann et al. The HER-2 overexpression was detected by IHC in 23 (29.5%) tumors in Hercep test (score 2+/3+) and in 24 (30.7%) in IRS scale (IRS 4–12). The overexpression of HER-2 was associated with poorly differentiated tumors, but this correlation was not significant (*P* = 0.064). No relationship was found between HER-2 expression and primary tumor size and degree of spread to regional lymph nodes. Both univariate and multivariate analyses revealed that TNM stage and patient's age were the crucial negative prognostic factors. No correlation was observed between patient survival and expression of HER-2 estimated using both scales. This research did not confirm HER-2 expression (evaluated with immunohistochemistry) value as a prognostic tool in GC.

## 1. Introduction

Gastric cancer (GC) is one of the most common neoplasms in the world. About 930 000 new cases are estimated to occur annually [1]. Despite the rapid development of several anticancer drugs and the identification of many prognostic and predictive factors, advanced GC is still strongly associated with a poor outcome with a median survival of 7–10 months in patients with metastatic or unresectable disease [2]. So far, the most important factor in prognosis and prediction for gastric cancer patients is the TNM stage, which is determined by primary tumor size, degree of spread to regional lymph nodes, and distant metastases. However, in patients with the same stage, prognosis could be various, so further studies are necessary to develop new prognostic factors.

HER-2 (*human epidermal growth factor receptor 2*) is a transmembrane tyrosine kinase receptor involved in development and progression of various solid tumor types such as breast cancer, pulmonary adenocarcinoma, and colorectal and gastric cancer [3–5]. Although a ligand for HER-2 has not been identified, recent studies suggest that HER-2 is the preferred heterodimerization partner for other members of the epidermal growth factor receptors family. The tyrosine kinase activity of HER-2 intracellular domain triggers signal transduction pathways, which are involved in cell proliferation, migration, apoptosis, and differentiation [6].

Although trastuzumab is currently approved for treatment of HER-2 overexpressing breast cancer [7] and HER-2 overexpressing metastatic gastric cancer as a result of ToGA trial [8], there are conflicting results in studies of HER-2 immunoreactivity and its relationship to prognosis on gastric cancer patients. However, according to ToGA trial data amplification was not sufficient enough to reliably detect the patients that had a significant benefit from trastuzumab therapy. IHC is then more predictive than FISH. Some researchers have reported that HER-2 overexpression or amplification is strongly associated with a poor outcome in gastric cancer [9–11], but other studies have failed to find any association with the prognosis [12, 13]. In this study, the expression of HER-2 in gastric cancer was investigated by immunohistochemistry. The correlations between HER-2 parameters of expression and clinicopathological parameters and overall survival were analyzed.

## 2. Methodology

### 2.1. Patients and Tissue Specimens

Seventy-eight patients with histologically confirmed adenocarcinoma of the stomach operated on with curative intent (R0) entered the study. The group of patients consisted of 54 males and 24 females with ages ranging between 37 and 84 years old (mean age: 62 years old). The stage of tumors was assessed according to the 5th ed. of TNM Classification of Malignant Tumors [14] (stage I: 25 patients (32%); II: 18 (23%); III: 17 (22%); IVM0: 18 (23.1%). According to Lauren's classification: 19 (24%) cases were intestinal, 44 (56.%) diffused and 15 (19.%) were of a mixed type. All patients underwent elective total gastrectomy and D2 lymphadenectomy with curative intent (the mean number of dissected lymph nodes was 19). The adjuvant chemotherapy was administered in 53 cases of tumors infiltrating beyond the muscularis propria or in patients with lymph node involvement. The followup was scheduled every 3 months for the first 2 years and then every 6 months. Chest X-ray, abdominal sonography, CT scan as well as clinical and endoscopic examinations were performed. The information about overall survival were obtained from Lower-Silesian Regional Cancer Registry. The data were collected in a retrospective manner.

The tissue samples were fixed in 10% buffered formalin and embedded in paraffin. In each case, hematoxylin- and eosin-stained preparations were subjected to histopathological evaluation by two pathologists.

### 2.2. Immunohistochemistry

Formalin-fixed, paraffin embedded tissue was freshly cut (4 *μ*m). The sections were mounted on superfrost slides (Menzel Gläser, Germany), dewaxed with xylene, and gradually hydrated. The activity of endogenous peroxidase was blocked by 5 min exposure to 3% H_2_O_2_. All the studied sections were boiled for 15 min at 250 W in the Antigen Retrieval Solution (DakoCytomation, Denmark). Then, immunohistochemical reactions were performed using the rabbit antihuman antibody detecting HER-2 (optimally prediluted) (DakoCytomation, Denmark). The tested sections were incubated with antibodies for 1 h at room temperature. The subsequent incubations involved biotinylated antibodies (15 min, room temperature) and a streptavidin-biotinylated peroxidase complex (15 min, room temperature) (LSAB+, HRP, DakoCytomation, Denmark). NovaRed (Vector Laboratories, UK) was used as a chromogen (10 min, at room temperature). All the sections were counterstained with Meyer's hematoxylin. In every case, control reactions were included, in which specific antibody was substituted by the Primary Mouse Negative Control (DakoCytomation, Denmark).

### 2.3. Evaluation of Reaction Intensity

The intensity of immunohistochemical reactions was estimated independently by two pathologists. In doubtful cases a reevaluation was performed using a double-headed microscope and staining was discussed until a consensus was achieved. It is supposed that application of breast cancer scoring to gastric cancer may produce an up to 50% false-negative rate if IHC is used. Thus in order to evaluate the HER-2 expression, the Remmele scale (IRS) [15] modified by the authors and standardised Hercep test score modified for GC by Hofmann et al. were applied (Table 1) [16]. In IRS scale the intensity of color reaction and percentage of positive cells were taken into account. The score represented a product of points given for the evaluated characters and it ranged from 0 to 12 (Table 1). Cases with expression of 0 to 3 in IRS scale and with score 0 to 1+ according to Hofmann et al.'s criteria were treated as cases without overexpression (Table 2). It is well known that patients with gastric cancer have a heterogeneous HER-2 expression. Intratumoral heterogeneity of HER2 expression may potentially contribute to inaccurate assessment of HER2 status. There is evidence that tumor heterogeneity is more common in gastric cancer (4.8%) than in breast cancer (1.4%) [16]. We observed 9% cases with heterogenous HER-2 immunoreactivity. Therefore, in some institutions the evaluation of HER-2 expression in the immunohistochemical staining is carried out using several sections of tissue sample. In our study single slide from large representative resection specimen for each cancer case was analyzed and 10% cutoff for the number of reactive cells was retained.

### 2.4. Statistical Analysis

Statistical analyses were performed using the Statistica 9.1 software (StatSoft Inc., Tulsa, OK, USA) and R language and environment for statistical computing (R Foundation for Statistical Computing, Vienna, Austria, http://www.R-project.org/). Chi^2^ and Spearman rank correlation were used to analyze associations between immunohistochemical parameters of HER-2 expression and clinicopathological features. The overall survival rate was estimated by the Kaplan-Meier method. Multivariate analyses (Cox proportional hazard regression models) were also performed to assess the prognostic value of HER-2 expression and other clinicopathological features.

## 3. Results

### 3.1. HER-2 Expression in Gastric Cancer

**Figure 1:**
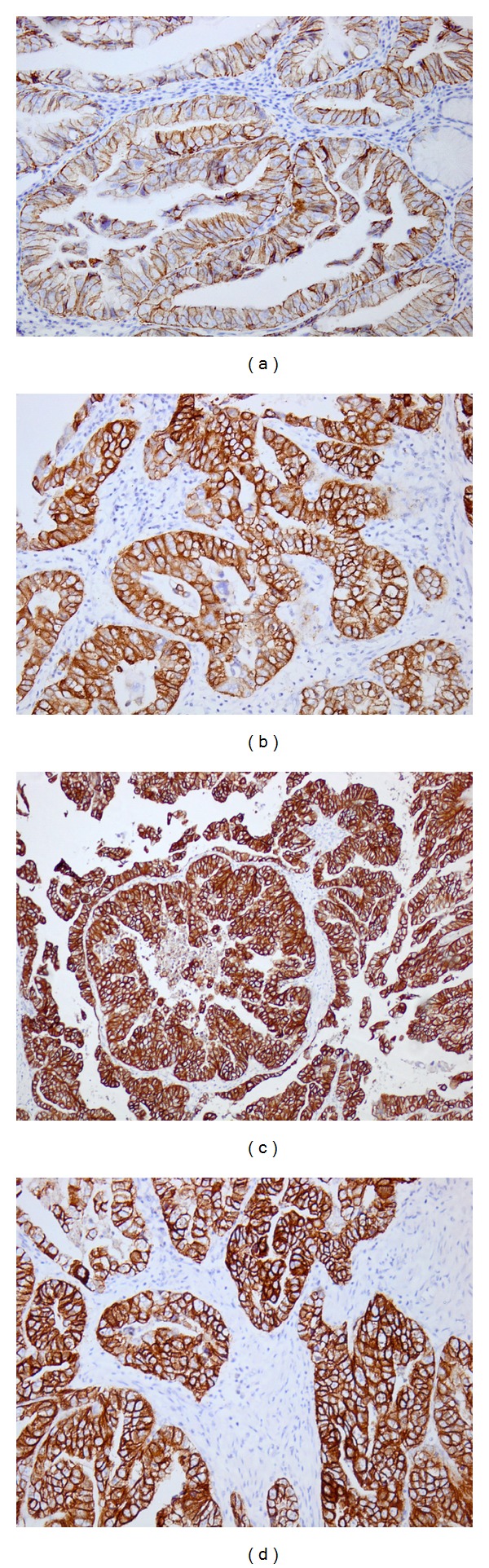
Immunohistochemical expression of HER-2 in gastric cancer tissue: (a) Hercept test: 1+, IRS: 4, ×200; (b) Hercep test: 2+, IRS: 8, ×200; (c) HercepTest: 3+, IRS: 12, ×200; (d) Hercep test: 3+, IRS: 12, ×200.

The HER-2 overexpression was detected in 23 (29.5%) tumors in Hercep test (IHC 2+/3+) and in 24 (30.7%) in IRS scale (IRS 4–12) (Figure 1). Lack of HER-2 expression was found in 35 cases (44.9%). In all samples membrane localization of HER-2 in gastric cancer cells was dominantly observed, but cytoplasmic topography was also described (Table 2).

### 3.2. Association between HER-2 Immunoreactivity and Clinicopathological Parameters

The overexpression of HER-2 was associated with poorly differentiated tumors, but this correlation was marginally significant (*P* = 0.064). Interestingly, the statistical analysis revealed a significant association between the percentage of HER-2 positive gastric cancer cells and the presence of ulceration in clinical material (*P* = 0.035). No relationship (Table 3) was found between HER-2 overexpression and primary tumor size, degree of spread to regional lymph nodes, type of Lauren's classification and inflammatory infiltration of the tumor (tumor infiltrating lymphocytes).

### 3.3. HER-2 Expression and Patients Survival

**Figure 2:**
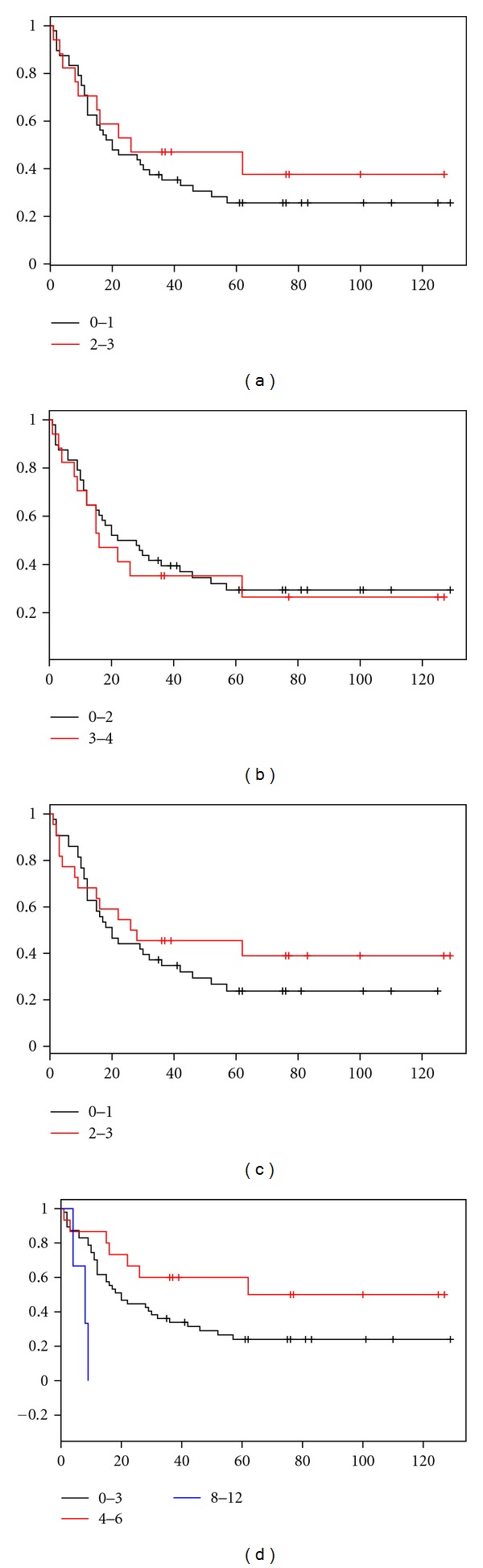
Kaplan-Meier curves for survival and expression of HER-2 in studied group of 78 gastric cancer patients: No correlation was observed between Hercep test score (*P* = 0.454) (a), percentage of HER-2 positive gastric cancer cells (*P* = 0.717) (b), intensity of immunohistochemical reaction (*P* = 0.397) (c) and IRS rate (*P* = 0.071) (d).

The survival analysis performed with Kaplan-Meier method revealed that tumor size at diagnosis (pT3, pT4), regional lymph nodes metastases, and patient's age covariated with negative prognostic significance (*P* = 0.028, *P* = 0.015 and *P* = 0.0048, resp.). No correlation was observed between patient survival and overexpression of HER2 (Figure 2). We only observed a tendency towards worse prognosis among 11 patients with highest HER-2 IRS (8–12 IRS); however, this correlation was not statistically significant (*P* = 0.071). The multivariate analysis (Table 4) confirmed the unfavorable prognostic significance of advanced age (*P* = 0.014), advanced pT stage (*P* = 0.027), nodal involvement (*P* = 0.027), and lack of prognostic value of HER-2 expression parameters.

## 4. Discussion

The study has described the expression of HER-2, detected by immunohistochemistry in invasive gastric cancer. The immunoreactivity of HER-2 did not reveal any correlation between histopathological parameters, such as Lauren's classification type or gastric cancer grading. Interestingly, only one significant relationship between the presence of ulceration and the percentage of positive HER-2 cancer cells was observed. The survival analysis did not confirm the prognostic significance of HER-2 overexpression in gastric cancer patients.

HER-2 reactivity has been studied extensively in gastric cancer in order to correlate it to clinicopathological features and prognosis, due to the potential using of trastuzumab as an adjuvant chemotherapy [3, 9–12]. In our previous study based on immunohistochemical evaluation of 396 breast cancer specimens, HER-2/neu overexpression has been documented in 18% of invasive cancer cases and has been associated with a poor prognosis [17]. Other authors documented HER-2 overexpression in 10–34% of breast cancer cases [4]. Favorable clinical results with anti-HER-2/neu therapy in breast cancer have led to the analysis of its expression in other solid tumors, such as ovarian, lung, colon and gastric cancers [5, 9, 11, 12, 18]. A number of studies have analyzed HER-2/neu immunoexpression in GC, but the clinical significance of its expression is not fully clear yet. Some authors documented that HER-2/neu appears to be an important prognostic factor in GC [3, 9, 12, 16], however, the literature is conflicting at this point, and other studies did not reveal any correlation between HER-2/neu overexpression and a poor prognosis [13, 19]. The rate of HER-2/neu positivity in GC is estimated to be between 15% and 20% [1, 3, 9, 12, 16, 20].

This study confirmed that parameters of TNM classification are the most important prognostic factors in gastric cancer. However, we did not observe a statistically significant correlation between HER-2 overexpression and Lauren's classification, which is also known as a significant prognostic factor. It was observed a distinct tendency between HER-2 expression and Lauren's type of cancer, because 40% of intestinal type is characterized by elevated level of HER-2 expression and only 20% of diffuse type were HER-2 overexpressed, but the difference was statistically not significant. Similar results were published by Gravalos and Jimenof [6], because they revealed relationship between HER-2 overexpression and Lauren's type, but this correlation was also statistically not significant. Interestingly, Kang et al. [21] reported that HER-2 overexpression correlated with the histological type according to Lauren's classification with statistical significance (34% intestinal type; 6% diffuse). Park et al. [9] having used FISH in the detection of HER-2 amplification observed that intestinal-type cancers were associated with a higher HER-2 amplification rate than diffuse-type cancers (*P* < 0.05).

Our research did not confirm the value of HER-2 overexpression in prognosis on GC patients. Similar results were observed by Tateishi et al. [13] and Sasano et al. [19], who did not find any relationship between HER-2 expression and prognosis. However, the vast majority of studies reported a direct correlation between HER-2 overexpression and GC patients survival. Park et al. [9] revealed that tumors with HER-2 amplification (demonstrated in FISH) exhibited poor mean survival. Surprisingly, the above mentioned study showed that HER-2 amplification was more common in the intestinal type of GC, which is usually associated with better prognosis. Staining intensity of HER-2 was also strongly correlated with unfavorable outcome in Zhang's et al. study. Survival curves, computed according to the method of Kaplan-Meier, showed that HER-2 overexpression in 102 gastric cancer patients was significantly correlated with decreased survival [11].

## 5. Conclusions

The significance of HER-2 overexpression in GC and its impact on survival is controversial. This research did not confirm value of HER-2 expression detected by immunohistochemistry as a prognostic tool in GC. In spite of this accurate assessment of HER-2/neu status is essential to determine which patients might benefit from the monoclonal antibody therapy. This study confirmed that parameters of TNM classification are the most important prognostic factors in gastric cancer.

##  Conflict of Interests

The authors declare that they have no conflict of interests.

## Figures and Tables

**Table 1:** Two procedures for evaluation of HER-2 expression.

Percentage of positive cells	IRS (Immunoreactive Score) modified by authors^a^		Points
	Points	Intensity of reaction	
No positive cells	0	No reaction	0
<25% positive cells	1	Weak colour reaction	1
25–50% positive cells	2	Moderate intensity	2
51–75% positive cells	3	Intense reaction	3
>75% positive cells	4		

Consensus recommendations on HER-2 scoring for gastric cancer^b^			

Definition			Score
No reactivity or membranous reaction in <10% of cells			0
Faint complete or partial membranous reactivity in >10% of cells			1+
Moderate complete or basolateral membranous reactivity in >10% of cells			2+
Strong complete or basolateral membranous reactivity in >10% of cells			3+

^a ^IRS score (Immunoreactive Score) according to Remmele and Stegner [15] modified by authors

^b^Hercep test criteria modified by Hofmann et al. [16] for gastric cancer.

**Table 2:** Parameters of HER-2 immunoreactivity.

Immunohistochemical parameters	Score/points	N (%)	Interpretation
Hercep test score^a^	0	35 (44.9)	Negative
	1+	20 (25.6)	Negative
	**2+**	**17 (21.8)**	**Overexpression**
	**3+**	**6 (7.7)**	**Overexpression**

Percentage of positive HER-2 cancer cells (%)^b^	0	35 (44.9)	
	1	12 (15.4)	
	2	10 (12.8)	
	3	10 (12.8)	
	4	11 (14.1)	

Intensity of HER-2 reaction (I)^c^	0	35 (44.9)	
	1	15 (19.2)	
	2	21 (26.9)	
	3	7 (9.0)	

HER-2 IRS^d^	0-1	42 (53.8)	Negative
	2-3	12 (15.4)	Negative
	**4–8**	**20 (25.6)**	**Overexpression**
	**9–12**	**4 (5.2)**	**Overexpression**

^a^Hercep test criteria modified by Hofmann et al. [16] for gastric cancer.

^b^Percentage of HER-2 positive gastric cancer cells (%) according to Remmele and Stegner [15] modified by authors.

^c^Intensity of immunohistochemical reaction in cancer cells (I) according to Remmele and Stegner [15] modified by authors.

^d^IRS score (Immunoreactive Score) according to Remmele and Stegner [15] modified by authors.

**Table 3:** Correlations between HER-2 expression and clinicopathological parameters.

Clinicopathological parameters	Hercep test^a,b^	HER-2%^a,c^	HER-2 I^a,d^	HER-2 IRS^a,e^
Tumor size (pT)	0.211	0.454	0.389	0.193
Nodal metastases (pN+)	0.945	0.736	0.702	0.750
Grading	**0.064**	0.212	0.189	0.202
Lauren's classification	0.505	0.835	0.692	0.554
Age^f^	0.570	0.935	0.693	0.693
Ulceration	0.672	**0.035**	0.162	**0.074**
Tumor infiltrating lymphocytes	0.839	0.889	0.717	0.852

^a^ *P* value of Chi^2^ test.

^b^Hercep test criteria modified by Hofmann et al. [16] for gastric cancer.

^c^Percentage of HER-2-positive gastric cancer cells (%) according to Remmele and Stegner [15] modified by authors.

^d^Intensity of immunohistochemical reaction in cancer cells (I) according to Remmele and Stegner [15] modified by authors.

^e^IRS score (ImmunoReactive Score) according to Remmele and Stegner [15] modified by authors.

^f^Spearman's rank correlation.

**Table 4:** Multivariate Cox proportional hazard regression analysis of HER-2 expression and clinicopathological parameters influence on patients overall survival.

Clinicopathological parameters	*P*-value
Age	**0.014**
Nodal metastases (pN)	**0.003**
Tumor size (pT)	**0.027**
Hercep test score^a^	0.775
Percentage of positive HER-2 cancer cells^b^	0.741
Intensity of HER-2 color reaction (I)^c^	0.859
HER-2 IRS^d^	0.321

^a^Hercep test criteria modified by Hofmann et al. [16] for gastric cancer.

^b^Percentage of HER-2-positive gastric cancer cells (%) according to Remmele et al. [15] modified by authors.

^c^Intensity of immunohistochemical reaction in cancer cells (I) according to Remmele and Stegner [15] modified by authors.

^d^IRS score (ImmunoReactive Score) according to Remmele and Stegner [15] modified by authors.
